# The Large Tegument Protein pUL36 Is Essential for Formation of the Capsid Vertex-Specific Component at the Capsid-Tegument Interface of Herpes Simplex Virus 1

**DOI:** 10.1128/JVI.02887-14

**Published:** 2014-11-19

**Authors:** Wan H. Fan, Ashley P. E. Roberts, Marion McElwee, David Bhella, Frazer J. Rixon, Rebecca Lauder

**Affiliations:** MRC—University of Glasgow Centre for Virus Research, Glasgow, United Kingdom

## Abstract

Herpesviruses have a characteristic particle structure comprising an icosahedral capsid, which contains the DNA genome and is, in turn, surrounded by a proteinaceous tegument layer and a lipid envelope. In herpes simplex virus, the interaction between the capsid and tegument is limited to the capsid vertices and involves two minor capsid proteins, pUL17 and pUL25, and the large inner tegument protein pUL36. pUL17 and pUL25 form a heterodimeric structure, the capsid vertex-specific component (CVSC), that lies on top of the peripentonal triplexes, while pUL36 has been reported to connect the CVSC to the penton. In this study, we used virus mutants with deletions in the genes for pUL36 and another inner tegument protein, pUL37, to analyze the contributions of these proteins to CVSC structure. Using electron cryomicroscopy and icosahedral reconstruction of mutants that express pUL17 and pUL25 but not pUL36, we showed that in contrast to accepted models, the CVSC is not formed from pUL17 and pUL25 on their own but requires a contribution from pUL36. In addition, the presence of full-length pUL36 results in weak density that extends the CVSC toward the penton, suggesting either that this extra density is formed directly by pUL36 or that pUL36 stabilizes other components of the vertex-tegument interface.

**IMPORTANCE** Herpesviruses have complex particles that are formed as a result of a carefully controlled sequence of assembly steps. The nature of the interaction between two of the major particle compartments, the icosahedral capsid and the amorphous tegument, has been extensively studied, but the identity of the interacting proteins and their roles in forming the connections are still unclear. In this study, we used electron microscopy and three-dimensional reconstruction to analyze virus particles formed by mutants that do not express particular interacting proteins. We show that the largest viral protein, pUL36, which occupies the layer of tegument closest to the capsid, is essential for formation of structurally normal connections to the capsid. This demonstrates the importance of pUL36 in the initial stages of tegument addition and provides new insights into the process of virus particle assembly.

## INTRODUCTION

The herpes simplex virus type 1 (HSV-1) virion is composed of an icosahedral capsid, which contains the DNA genome, surrounded by tegument and bounded by an envelope (1). The tegument is a proteinaceous layer of variable dimensions and composition that is characteristic of herpesviruses. In HSV-1 it contains more than 20 proteins (2), which are sometimes subclassified as components of an inner and outer tegument. The first stage of HSV-1 virion morphogenesis is the assembly of procapsids in the nuclei of infected cells (3, 4). The viral genome is packaged into the procapsid through a unique portal vertex (5–7). This process triggers a procapsid-to-capsid transition during which the capsid shell adopts a stable more angular structure (8, 9). DNA packaging is dependent on the presence of several capsid/packaging proteins, which either are minor components of the capsid or associate transiently with the portal during DNA packaging (10, 11). DNA-containing capsids (C-capsids) exit from the nucleus by budding through the nuclear envelope, which releases them into the cytosol (12). Tegument addition seems to be a multistep process (12–14). Proteins of the inner tegument, principally pUL36 and pUL37, which are closely linked with the capsid, appear to be added shortly after the capsid exits from the nucleus. The outer tegument proteins are thought to accumulate predominantly at vesicles derived either from the *trans*-Golgi network (TGN) or from endosomal membranes, where they associate with the viral glycoproteins. Envelopment occurs when the capsid plus inner tegument buds into these vesicles (15–18). The targeting of the outer tegument and envelope proteins to the cellular membranes does not depend on the presence of capsids, as L-particles, which consist of enveloped tegument, are still assembled under conditions where capsid formation is blocked (19, 20). Although the inner tegument proteins pUL36 and pUL37 are closely associated with capsids, they are also present in L-particles (20–22), and it has been established that pUL36 can target pUL37 to the TGN in the absence of capsids (23). Analysis of deletion mutants has shown that pUL36 and pUL37, which interact with each other (24, 25), are mutually codependent for incorporation into L-particles, suggesting that they normally occur as a complex in virus particles (20). In HSV-1, pUL36 and pUL37 are both essential for virion assembly, and virus mutants in which either gene has been deleted accumulate large numbers of nonenveloped capsids in the cytoplasm of infected cells (20, 26, 27).

Unlike the capsid, which has a defined structure, the tegument and envelope are highly variable among HSV-1 virions. This was illustrated by icosahedral reconstructions of frozen hydrated virions, which revealed the capsid but failed to show the bulk of the tegument and envelope (28), and by tomographic reconstruction which directly illustrated the nonuniform nature of the tegument and envelope (29, 30). The nature of the interaction between the capsid and tegument differs among the alpha, beta, and gamma herpesviruses (28, 29, 31–35), and in HSV-1 (an alphaherpesvirus), consistent interaction appears to be confined to the region around the capsid vertices. Comparison of three-dimensional (3D) reconstructions of intact HSV-1 virions and nuclear B-capsids revealed the presence of additional density at the capsid vertices in virions (28, 29). These star-shaped densities, which extend from the top of the penton to the adjacent triplexes and hexons, were originally considered to be tegument. The behavior and properties of pUL36 and pUL37 suggested that they act as a bridge linking the capsid and tegument, and pUL36 was initially proposed as the most likely contributor to this “tegument” material (28). Subsequently, several studies that examined nuclear C-capsids have reported that the minor capsid/DNA packaging proteins pUL17 and pUL25 make up part of this material and form the arms of the star (36–38). The pUL17/pUL25 density, which was initially designated the C-capsid specific component (CCSC), was later shown to be also present on A- and B-capsids and was consequently renamed the capsid vertex-specific component (CVSC) (11). It has also been shown to be present in capsids of another alphaherpesvirus, pseudorabies virus (PrV) (39). The locations of pUL17 and pUL25 were determined by reconstructing mutated forms that had protein tags inserted into defined positions (11, 37–39). This suggested that pUL25 formed the portion of the CVSC located distal to the capsid vertex, with pUL17 making up the proximal component. Analysis of the protein composition of the nuclear capsids examined in these studies suggested that pUL36 did not contribute to the CVSC density (38). However, a subsequent study, which examined chemically stripped virions, reported that pUL36 did contribute to the larger density seen in virions, where it formed the hub of the star occupying a position over the pentons (40). More recently, high-resolution reconstruction of mature gammaherpesvirus (Kaposi's sarcoma-associated herpesvirus [KSHV]) virions has produced a different interpretation by revealing that the distal part of the archetypal CVSC that sits above the two triplexes (Ta and Tc in reference 41) is formed by the KSHV homologue of pUL17, with the pUL25 homologue occupying a position on top of the penton (35). This essentially inverts the positions of these two proteins from those proposed in the earlier studies.

Although much recent work has gone into clarifying the roles of pUL17, pUL25, and pUL36 in forming this star-shaped density, questions remain over their spatial organization and the nature of their interactions. In this study, we further investigated the contributions of pUL36 and pUL37 by examining the structure and composition of capsids produced by UL36-null and UL37-null mutants.

## MATERIALS AND METHODS

### Cells and viruses.

Baby hamster kidney cells (BHK-21), control and pUL34-expressing Vero cells (143/1099E), and rabbit skin (RS) cells expressing pUL36 (HAUL36) and pUL37 (80CO2) were cultured as described previously (20, 42). Briefly, cells were grown at 37°C in either Dulbecco's modified Eagle medium (DMEM; Gibco-Invitrogen) supplemented with 10% fetal calf serum (FCS; Gibco-Invitrogen) or Glasgow minimum essential medium (GMEM; Gibco-Invitrogen) supplemented with 10% newborn calf serum (NCS; Gibco-Invitrogen) and tryptose phosphate broth (TPD; E & O Laboratories).

Wild-type HSV-1 (strain 17^+^) was propagated on BHK-21 cells. The UL36-null (ARΔUL36 and KΔUL36), UL37-null (FRΔUL37), and UL34-null (vRR1072) mutants were propagated on HAUL36, 80CO, and 143/1099E cells, respectively (20, 42). Cells were infected at 0.01 PFU per cell and incubated until the cells detached from the substrate. Virions were concentrated from the culture medium by centrifugation at 17,000 × *g* for 2 h.

### Antibodies.

The following antibodies against HSV-1 structural proteins were used: mouse monoclonal antibody (MAb) DM165 against the major capsid protein pUL19 (VP5) (43); MAb203 and MAb166 against the CVSC proteins pUL17 and pUL25, respectively (44); MAbE12-E3 against the inner tegument protein pUL36 (45); and rabbit polyclonal antibody M780 against the inner tegument protein pUL37 (46). Horseradish peroxidase-conjugated goat anti-mouse (GAM_HRP_) and goat anti-rabbit (GAR_HRP_) antibodies were from Sigma.

### Capsid purification.

Capsids were prepared essentially as described by Roberts et al. (20), except that the cells were harvested after 16 h of incubation at 37°C.

### Western blot analysis.

Protein samples were separated by electrophoresis on 10% sodium dodecyl sulfate (SDS)-polyacrylamide gels. Bands were transferred to Hybond ECL nitrocellulose membranes (GE Healthcare) using a semidry transfer station (Bio-Rad). Blots were blocked overnight with 5% milk powder (Sigma) in TBS-Tween (0.02 M Tris-HCl [pH 8], 0.15 M NaCl, 1% Tween 20) and then incubated in the appropriate antibodies diluted in TBS-Tween. Detection of proteins was through enhanced chemiluminescence (ECL; GE Healthcare) on medical X-ray film (Fuji).

### Electron microscopy.

Thirty-five-millimeter dishes of cells were fixed with 2.5% glutaraldehyde and 1% osmium tetroxide. Fixed cells were harvested and pelleted through 1% SeaPlaque agarose (Flowgen). The cell pellets were dehydrated through a graded alcohol series and embedded in Epon 812 resin. Thin sections were cut and examined in a JEOL 1200 EX II electron microscope.

### Data acquisition and 3D image reconstruction of purified capsids.

Purified capsids (3 μl) were applied to holey carbon grids (Quantifoil) coated with a thin carbon film and then were plunged into liquid ethane cooled in a liquid nitrogen bath using a Vitrobot (FEI Eindhoven). Grids were viewed at <100 K on a JEM2200 FS electron microscope equipped with a Gatan 626 cryostage. Energy-filtered images were collected at a slit width of 20 eV at a magnification of ×40,000 and an electron dose of ∼8 e^−^/A^2^ using an Ultrascan 4000 charge-coupled-device (CCD) camera (Gatan). Micrographs were recorded using Digital Micrograph software (Gatan) at a sampling rate determined at 2.66 Å/pixel. Screening, particle picking, and contrast transfer function (CTF) correction were carried out using the Bsoft image processing package (47). Each micrograph was visually assessed for aberrations. The defocus of selected micrographs was estimated and individual particles were picked. CTF correction was performed and particle images were prepared for 3D reconstruction using SPIDER to removing low-frequency gradients, center particles, and invert their contrast (48). PFT2 was used to determine and refine the orientation and origin of individual particles through iterative projection matching (49). The particle images were then used to generate a 3D reconstruction using EM3DR2 (50, 51). The refinement process was iterated until the calculated resolution of the resulting map no longer improved. Resolution assessment was performed by dividing the data into two equal subsets from which two reconstructions were calculated; a fuzzy mask was applied such that only density between radii of 450 and 590 Å from the origin was analyzed (capsid and tegument layers). Resolution was estimated by Fourier shell correlation (FSC) with a cutoff of 0.5. The map resolutions and numbers of particles used for each reconstruction are listed in Table 1. 3D reconstructions were visualized using the UCSF Chimera software package (52). Unless otherwise stated, all capsids are shown at a contour level above the mean plus one standard deviation of the total map density (+1 σ).

**TABLE 1:** Numbers of capsids selected for each virus and resolution of each reconstruction

Virus	Type of capsid	Resolution (Å)	No. of particles
WT	Cytoplasmic	21	1,149
	Nuclear	24	475
ARΔUL36	Cytoplasmic	18	3,258
	Nuclear	23	1,056
KΔUL36	Cytoplasmic	19	2,608
FRΔUL37	Cytoplasmic	18	2,132
	Nuclear	22	658
vRR1072	Nuclear	21	836

### Comparisons of mutant capsids with the HSV-1 B-capsid.

The differences in the densities were observed by superimposing mutant capsid reconstructions onto an HSV-1 B-capsid map. The HSV-1 B-capsid reconstruction was rendered from the EMDB-5660 map published by Homa et al. (39). A low-pass filter from the EMAN image processing program proc3d (53) was used to filter the B-capsid map to 21 Å. The resulting map was surface contoured and colored gray. Radially colored, mutant capsid maps, also generated at 21 Å, were superimposed onto the B-capsid reconstruction and visualized using UCSF Chimera.

### Segmentation of mutant capsid reconstructions.

Each capsid reconstruction was computationally segmented using the UCSF Chimera extension Segger (54) at three threshold settings (+1 σ, +0.5 σ, and +0.2 σ). One set of triplexes (Ta and Tc), two penton subunits, and the vertex-associated (CVSC) density were manually selected and colored from each reconstruction and for each threshold setting. All remaining segments were deleted. Isolated structures were visualized using UCSF Chimera.

## RESULTS

### Formation of the archetypal CVSC requires the presence of the inner tegument protein pUL36.

**FIG 1:**
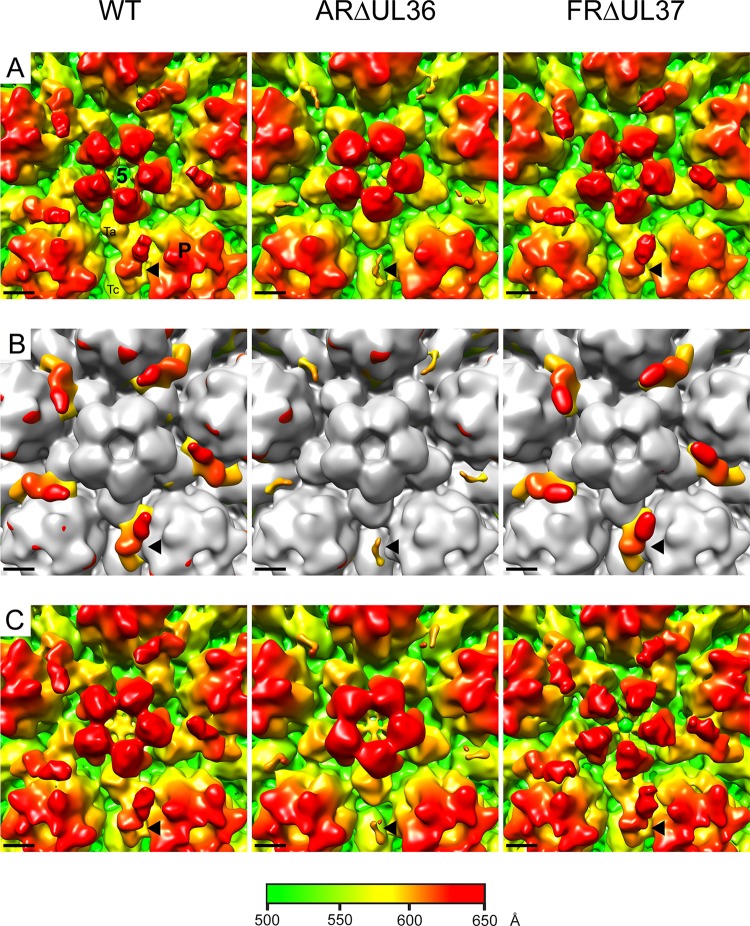
Comparison of CVSC densities in HSV-1 WT and mutant virus capsids. A region around the vertex (indicated by the white square in Fig. S1B in the supplemental material) of icosahedrally reconstructed C-capsids purified from the cytoplasm (A and B) or nucleus (C) of cells infected with WT HSV-1 or the inner tegument deletion mutants ARΔUL36 and FRΔUL37 is shown. Panels A and C are shown radially colored. Panel B shows the cytoplasmic C-capsid maps in panel A superimposed on a B-capsid map (shown in gray). The locations of the penton (5), one of the five peripentonal hexons (P), and a Ta and Tc triplex are marked on the WT image in panel A. Each black arrowhead indicates the position of one CVSC density. Scale bar = 50 Å.

After they have left the nucleus, cytoplasmic C-capsids become tegumented and enveloped to form mature virions. To analyze the sequence of events occurring during tegument addition, we determined the structures of cytoplasmic C-capsids prepared from BHK cells infected with wild-type (WT) HSV-1 and two inner tegument mutants with complete deletions of the UL36 (ARΔUL36) and UL37 (FRΔUL37) genes (20). Previously, we reported problems with isolating cytoplasmic C-capsids from cells infected for 24 h with WT and FRΔUL37 due to aggregation of the partially tegumented capsids (20). To overcome these problems, we prepared capsids at 16 h after infection, when the concentration of cytoplasmic capsids was lower. C-capsids were isolated on sucrose gradients, and electron cryomicroscopic images were collected. The majority of the capsids in these preparations were C-capsids, which could be readily distinguished from contaminating A- and B-capsids, which lack DNA (see Fig. S1A in the supplemental material). 3D reconstructions were generated to final resolutions of 20 Å for the WT and 18 Å for ARΔUL36 and FRΔUL37. As expected, the structures of all three showed typical capsid features with hexons, pentons, and triplexes arranged on a T=16 icosahedral lattice, and no differences were apparent between them other than at the capsid vertices, which are known to be sites of interaction between capsid and tegument (Fig. 1A; see also Fig. S1B). Comparison with the nuclear B-capsid revealed additional densities on the outer surface of the capsids occupying the space between the P-hexons that surround the penton (Fig. 1B). The WT and FRΔUL37 structures closely resembled each other, with relatively extensive additional densities that are arranged in a radial pattern around the vertices. In contrast, the additional densities in ARΔUL36, although occupying similar positions, were much smaller. The presence of additional “star-shaped” densities at the vertices of intact virions is well established (28), and they are thought to be composed of the minor capsid/packaging proteins pUL17 and pUL25 and the inner tegument protein, pUL36. pUL36 has been reported to account for the inner, penton-capping hub of the star (40), with pUL17 and pUL25 making up the CVSC, which corresponds to the outer arms of the star (36, 39). Although the vertex-associated densities of the WT and FRΔUL37 cytoplasmic C-capsids were similar to the archetypal CVSC previously described for WT nuclear C-capsids (11, 36–38), those present in ARΔUL36 were significantly smaller. For convenience, we refer here to all forms of vertex-associated additional density as CVSC.

**FIG 2:**
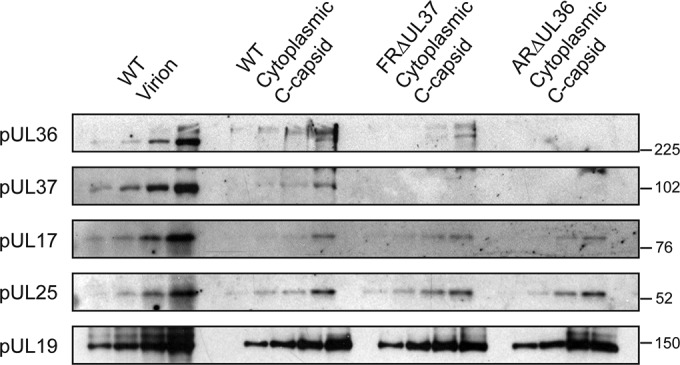
Protein compositions of HSV-1 WT and mutant virus capsids as determined by Western blot analysis of cytoplasmic C-capsids purified from the cytoplasm of cells infected with WT HSV-1 or the inner tegument deletion mutants ARΔUL36 and FRΔUL37. Serial 2-fold dilutions of each sample were run on a 10% polyacrylamide gel and transferred to Hybond ECL nitrocellulose membranes for analysis. Equal amounts of purified WT virions were loaded for comparative purposes. Proteins were visualized using antibodies MAbE12-E3 (pUL36), M780 (pUL37), MAb203 (pUL17), and MAb166 (pUL25). The major capsid protein pUL19 was monitored as a loading control using antibody DM165. The positions of molecular weight standards (in thousands) are indicated to the right of each panel.

pUL17 and pUL25, which make up the CVSC, are essential for packaging and retention of DNA in capsids. Therefore, the greatly reduced size of the CVSCs found on ARΔUL36 capsids was surprising, as both the component proteins would be expected to be present on DNA-containing C-capsids. The small size of the CVSC in these cytoplasmic C-capsids suggests either that the archetypal CVSC seen on WT nuclear C-capsids contains, or is stabilized by, pUL36 or that the UL36 mutant capsids contain altered amounts of pUL17 and pUL25. To investigate the latter possibility, we examined the protein composition of the capsids by Western blotting (Fig. 2). Increasing amounts of each capsid preparation were probed with antibodies against pUL17, pUL19, pUL25, pUL36, and pUL37. As expected, pUL36 and pUL37 were not detected in the ARΔUL36 and FRΔUL37 samples, respectively. ARΔUL36 capsids also lacked pUL37, thereby confirming that complex formation with pUL36 is required for addition of pUL37 to capsids (55). pUL36 and pUL37 were both present in WT capsids, but the amounts were appreciably lower than in WT virions. Importantly, the amounts of pUL17 and pUL25 in ARΔUL36 capsids were similar to those for the WT and FRΔUL37, thereby demonstrating that the reduced size of the CVSC was not caused by loss of either of these proteins.

**FIG 3:**
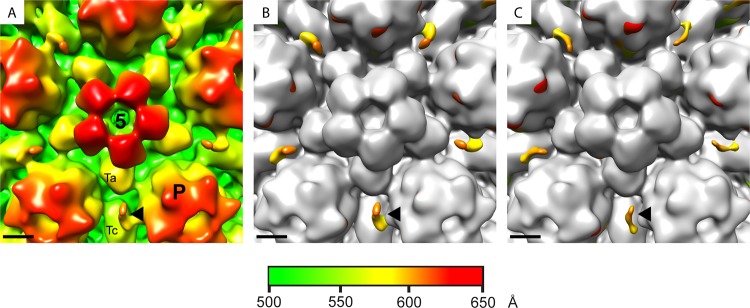
CVSC densities on capsids of two unrelated UL36 deletion mutants. The icosahedrally reconstructed structure of C-capsids purified from the cytoplasm of cells infected with the UL36 deletion mutant KΔUL36 is shown radially colored (A) and superimposed on a B-capsid map (shown in gray) (B). To allow comparison, the superimposed ARΔUL36/B-capsid map from Fig. 1B is shown again as panel C. The locations of the penton (5), a peripentonal hexon (P), and a Ta and Tc triplex are marked on panel A. Each black arrowhead indicates the position of one CVSC density. Scale bar = 50 Å.

To confirm that the smaller CVSC was directly related to the lack of pUL36, the experiment was repeated using an independent HSV-1 strain KOS mutant, KΔUL36 (26). KΔUL36 has an internal deletion which removes 3,600 bases from an internal region of the UL36 open reading frame. This incomplete deletion allows the expression of the N-terminal 361 amino acids of pUL36 (20, 26). Despite this difference, the phenotype of KΔUL36 resembles that of the complete deletion in ARΔUL36, with accumulation of cytoplasmic C-capsids but no progression to envelopment or formation of mature virions. KΔUL36 cytoplasmic C-capsids were prepared, imaged, and reconstructed to a final resolution of 19 Å using the conditions and procedures described above. Comparison with the WT B-capsid again showed additional densities at the vertices which were similar to those in ARΔUL36 and much smaller than those in WT or FRΔUL37 C-capsids (Fig. 3). The close similarities between the KΔUL36 and ARΔUL36 structures implies that the unexpectedly small size of their CVSCs is not mutant or virus strain specific but is directly related to the absence of full-length pUL36.

### Structural comparison of CVSCs from nuclear and cytoplasmic capsids.

**FIG 4:**
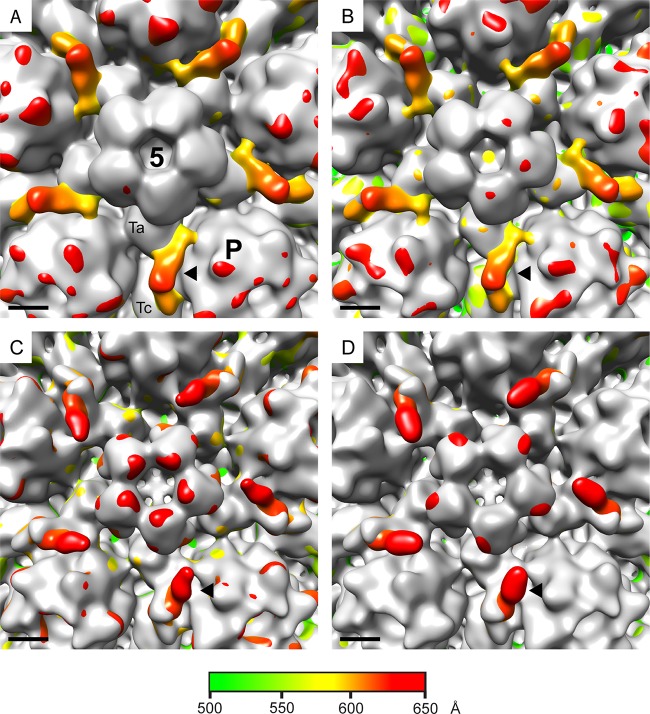
C-capsids confined to the nucleus have an intermediate-size CVSC. C-capsids purified from the nuclei of cells infected with the UL34 deletion mutant vRR1072 were icosahedrally reconstructed. Superimposed maps are shown between the vRR1072 map and those of a B-capsid (A) and cytoplasmic C-capsids of ARΔUL36 (B), the WT (C), and FRΔUL37 (D). The B-capsid and ARΔUL36 maps are shown in gray in panels A and B, respectively, while the vRR1072 map is shown in gray in panels C and D. The excess densities that are shown radially colored belong to vRR1072 (A and B), the WT (C), and FRΔUL37 (D). The locations of the penton (5), a peripentonal hexon (P), and a Ta and Tc triplex are marked on panel A. Each black arrowhead indicates the position of one CVSC density. Scale bar = 50 Å.

The apparent requirement for pUL36 was unexpected, since it was not believed to contribute to the CVSC density (38, 39). As this analysis was carried out on cytoplasmic capsids, one possibility is that the CVSC becomes destabilized during passage of capsids across the nuclear envelope and reforms in the cytoplasm only if pUL36 is present. To determine whether this was the case, nuclear C-capsids were prepared and reconstructed to final resolutions of 24 Å for the WT and ARΔUL36 and 22 Å for FRΔUL37. In each case the structure recapitulated that seen for cytoplasmic C-caspids, with normal-size CVSCs in the WT and FRΔUL37 but a smaller CVSC in ARΔUL36 (Fig. 1C). Thus, it appears that the effects seen in this study are not related to the subcellular origin of the capsid but are intrinsic to the mutants analyzed. These results imply that the presence or absence of pUL36 has a direct effect on the nature of the CVSC density and, in addition, that this effect is realized in both the nucleus and the cytoplasm. However, the role of the inner tegument proteins in maturation of nuclear capsids remains controversial, and the possibility of cross-contamination with capsids from the cytoplasm cannot be ruled out. Such contamination would not be unexpected given the relative crudity of the method used for nuclear isolation. Indeed, cosedimentation of WT and FRΔUL37 cytoplasmic capsids with isolated nuclei had been reported to account for the poor yields seen in previous analyses (20). To investigate this, we employed a gene UL34 deletion mutant of HSV-1 (vRR1072) (42). Together with its binding partner pUL31, pUL34 is essential for primary envelopment of capsids. Capsid assembly and DNA packaging occur normally in the absence of pUL34, but the capsids are retained in the nucleus and have no exposure to cytoplasmic modifications (42). Both pUL31 and pUL34 become transiently associated with perinuclear virions during nuclear egress but are not components of the mature virion (56, 57). To confirm the block on nuclear egress, we examined the distribution of capsids in thin sections of infected cells. Cells were infected at 37°C with 5 PFU/cell of vRR1072 for 20 h and then fixed and prepared for electron microscopy as described previously (20). As expected, capsids were present in both the nucleus and cytoplasm in UL34-expressing Vero (143/1099E) cells (see Fig. S2A in the supplemental material) but were seen only in the nucleus in control Vero (see Fig. S2B) and BHK (see Fig. S2C) cells. Therefore, C-capsids were isolated from the nuclei of vRR1072-infected BHK cells and reconstructed to a final resolution of 22 Å. As with all the other types of C-capsid described here, vRR1072 capsids had CVSC density around the vertices. In this case, the density was intermediate in size between the archetypal WT and FRΔUL37 CVSCs and the small CVSC of ARΔUL36. This is clearly illustrated by the maps shown in Fig. 4. The appearance of the CVSC after superimposition of the vRR1072 map on those of the B-capsid (Fig. 4A) and the ARΔUL36 capsid (Fig. 4B) was identical, reflecting the small size of the ARΔUL36 CVSC density, which is completely contained within that of vRR1072. Similarly, the maps of vRR1072 compared with those of the WT (Fig. 4C) and FRΔUL37 (Fig. 4D) are essentially the same. However, in these cases the WT and FRΔUL37 CVSCs are considerably more extensive than that present in vRR1072, with the additional densities representing the proximal part of the CVSC, which extends toward the penton.

**FIG 5:**
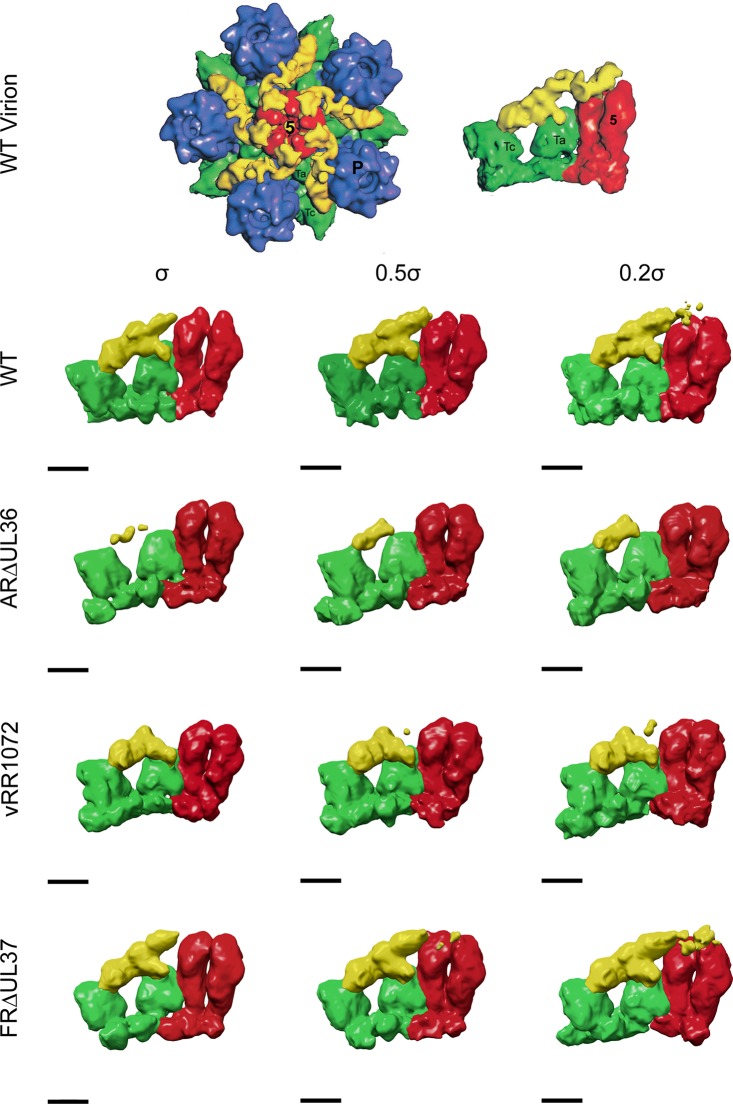
CVSC density on WT and FRΔUL37 capsids is threshold sensitive. The WT virion maps are reproduced from Fig. 4 of reference 28. The original images have been flipped 180° to show them in the correct hand. The left side shows one vertex from an icosahedrally reconstructed WT HSV-1 virion map, comprising the penton (red), surrounding P hexons (blue) and Ta and Tc triplexes (green), and the star-shaped CVSC/tegument density (yellow). The right side shows an enlarged side view of one CVSC/tegument density with two associated penton subunits and triplexes Ta and Tc. Equivalent regions from the WT, ARΔUL36, vRR1072, and FRΔUL37 cytoplasmic C-capsid maps are shown similarly colored for comparison. The C-capsid maps are shown at thresholds above the mean + 1 σ, mean + 0.5 σ, and mean + 0.2 σ (from left to right, respectively). Scale bar = 50 Å.

As has been noted previously, the icosahedrally ordered vertex densities seen in reconstructions of intact virions are not large enough to account for all the mass of the tegument proteins that are thought to be involved (28, 40). The presence of less ordered parts of the proteins, which are evident in density maps of the capsid vertices (see Fig. S3 in the supplemental material), makes the appearance of the CVSC as displayed by surface representation sensitive to the threshold at which they are viewed. Lowering the density threshold from the mean + 1 σ to the mean + 0.2 σ had little effect on the appearance of the ARΔUL36 (Fig. 5) and KΔUL36 (data not shown) CVSCs, which remained small and never approached the size of the archetypal CVSC seen on nuclear C-capsids (36). In contrast, reducing the threshold of the WT and FRΔUL37 capsid maps resulted in large increases in the size of the CVSC, which extended much further in the direction of the penton (Fig. 5). Interestingly, this was not the case for vRR1072 capsids, where the CVSC showed only a marginal increase in size at the lower threshold (Fig. 5).

## DISCUSSION

Despite their importance for virion assembly, the precise role of the inner tegument proteins, pUL36 and pUL37, in capsid maturation remains unclear. Their possible association with capsids inside the nucleus is uncertain, as is their likely role in nuclear egress (20, 26, 27, 58–60), although mutant studies have shown that DNA-containing capsids can move to the cytoplasm in the absence of pUL36 and pUL37. Both pUL36 and pUL37 are essential for virion assembly in HSV-1 and play a central role in the interaction between capsid and tegument. The initial aim of this study was to use virus mutants with deletions of UL36 and UL37 to investigate the contribution of these proteins to the ordered tegument density at the capsid vertices, which represents the only structurally resolvable link between these two virion compartments. Previous analyses appeared to show that pUL17 and pUL25 formed the density (CVSC) that is present on nuclear C-capsids (36–39, 61). pUL36 was thought to be involved only in virions, where it occupied a position overlying the penton adjacent to the region occupied by the CVSC (40). However, all previous studies have used WT viruses that express functional forms of these two proteins. In order to confirm this interpretation, we analyzed virus mutants with deletions of UL36 and UL37.

The small size of the CVSCs seen in multiple reconstructions of C-capsids from ARΔUL36 and the unrelated mutant KΔUL36 was surprising for two reasons: it was not large enough to accommodate the combined masses of its reported constituents, pUL17 and pUL25, and it suggested that pUL36 did play a role in forming this structure. Furthermore, the small CVSC was observed in reconstructions of both nuclear and cytoplasmic C-capsids, which additionally implied that pUL36 interacts with capsids in the nucleus as originally proposed by Bucks et al. (59). Since the ARΔUL36 cytoplasmic C-capsids contained normal amounts of pUL17 and pUL25, the difference in CVSC conformation could not simply be attributed to changes in its protein composition. Work by Leelawong et al. (62) suggests a possible explanation for this behavior. They reported that in PrV, a truncated form of pUL36 enters the nucleus, binds to the capsid, and facilitates capsid egress to the cytoplasm. On leaving the nucleus, the truncated isoform is replaced on capsids by full-length pUL36, which directs the cytoplasmic stages of virion assembly. The form associated with nuclear capsids was identified as a C-terminal fragment by using specific green fluorescent protein (GFP) tags and was mapped as corresponding to the ∼1,077 C-terminal residues. The C-terminal region of pUL36 is known to contain binding sites for pUL25 (63, 64), and pUL25 was shown to be required for binding of the pUL36 C-terminal fragment to capsids (62). Assuming that pUL36 in HSV-1 behaves in a fashion similar to that of its PrV counterpart, an equivalent C-terminal fragment would be expected to enter the nucleus and bind to capsids before being displaced by full-length pUL36 following egress to the cytoplasm. Such a fragment would be difficult to identify in stained protein profiles of the HSV-1 nuclear capsids, which may explain why its possible role in the CVSC was not suspected. Under these circumstances, the C terminus of pUL36 could contribute directly to the ordered density of the CVSC or, by binding to pUL25, could stabilize the conformation of the pUL17-pUL25 complex, making the CVSC more readily visualized in icosahedrally ordered reconstructions. This would explain the relatively large CVSC density seen with capsids from viruses that express full-length pUL36 (WT, FRΔUL37, and vRR1072) compared to that seen with viruses that lack the pUL36 C-terminal sequences (ARΔUL36 and KΔUL36). As mentioned earlier, KΔUL36 has an incomplete deletion of UL36 and retains the 361 N-terminal codons of UL36 (26). The retained UL36 sequences are expressed as a 43-kDa fragment that was shown to bind to cytoplasmic capsids (20). However, the close similarity between the structures of ARΔUL36 and KΔUL36 capsids shows that these N-terminal sequences do not detectably influence the CVSC density. This is consistent with the hypothesis that only the C-terminal sequences of pUL36 are involved and suggests that the N-terminal region is not icosahedrally ordered or is interacting with a different part of the capsid.

The results described here raise questions regarding previous interpretations of the nature of the CVSC. The small size of the density seen on ARΔUL36 and KΔUL36 capsids indicates that pUL17 and pUL25 alone do not account for the published structures and generates uncertainty over the relative positions and arrangement of these two proteins. It is interesting, therefore, that a recent study on KSHV virions reported a significantly different interpretation of the penton associated density than that previously suggested for HSV and PrV (36–39, 61). By fitting the crystal structure of HSV-1 pUL25 (65) into their KSHV virion map, Dai et al. (35) were able to establish that the KSHV homologue of pUL25 formed a globular density bound to the penton. Furthermore, good correlation between the predicted secondary structure of the pUL17 homologue and their density map strongly indicated that the pUL17 homologue formed the entire triplex binding density. The two globular densities were connected by a stalk region formed by aligned helices originating from the N-terminal sequences of pUL25 and from an unidentified third protein. Given the influence of pUL36 on the appearance of the CVSC, which we describe here, it seems highly probable that these unidentified stalk helices derive from pUL36, most likely from the C-terminal region, which is known to interact with pUL25 (62–64). Although our results show that pUL36 is not needed for pUL17 and pUL25 to bind to C-capsids, it is reasonable to speculate that interaction between the pUL25 and pUL36 stalk helices stabilizes the structure of the bound complex and allows it to be visualized.

For WT and FRΔUL37 cytoplasmic C-capsids, altering the threshold at which the reconstructions are displayed caused major changes to the appearance of the CVSC, which extended further toward the penton. The convergence of the WT and FRΔUL37 capsid maps toward the pattern seen with intact virions (28) suggests that these capsids contain additional material, which is present in virions but missing in the other capsid types analyzed. The obvious candidate is full-length pUL36, which was present on both WT and FRΔUL37 capsids. The region of the CVSC proximal to the penton typically appears as weak density, suggesting that it is relatively disordered. It is probable that further interactions with pUL36 (60, 64), which has been reported as forming a cap over the penton (40), are involved in stabilizing it. The lack of precision in the maps makes it impossible to determine whether pUL37 also contributes to this feature, but the overall similarity between the WT and FRΔUL37 maps suggests that any contribution would be small.

The structures of the mutant and WT capsids described here provide a working model for the initial stages of tegument-capsid interaction. Thus, pUL17 and pUL25 bind to the capsid inside the nucleus to form a proto-CVSC (as seen on ARΔUL36 and KΔUL36 capsids), most of which is not arranged in an icosahedrally consistent manner. DNA-containing capsids of this form can leave the nucleus and enter the cytoplasm. However, in the presence of a functional UL36 gene, nuclear capsids containing the proto-CVSC interact with a C-terminal fragment of pUL36 to produce the more stable CVSC structure seen in vRR1072 capsids. These capsids then enter the cytoplasm, where the C-terminal pUL36 fragment is displaced by full-length pUL36 to produce the larger, and threshold sensitive, CVSC seen in WT and FRΔUL37 C-capsids. WT C-capsids then undergo further tegumentation and envelopment, which results in the formation of the star-shaped vertex density seen in intact virions. To confirm this model and determine whether pUL36 actually forms part of the CVSC will require higher-resolution maps capable of revealing the structural signatures of the individual proteins involved.

## Supplementary Material

Supplemental material
