# Supplementary material for: The Large Tegument Protein pUL36 Is Essential for Formation of the Capsid Vertex-Specific Component at the Capsid-Tegument Interface of Herpes Simplex Virus 1
Source: J Virol. 2014 Nov 19;89(3):1502–11. doi: 10.1128/JVI.02887-14 (PMC4300765; doi:10.1128/JVI.02887-14)
Supplement: Supplemental material [file supp_89_3_1502__index.html]

The Large Tegument Protein pUL36 Is Essential for Formation of the Capsid Vertex-Specific Component at the Capsid-Tegument Interface of Herpes Simplex Virus 1 — Supplemental material 

# The Large Tegument Protein pUL36 Is Essential for Formation of the Capsid Vertex-Specific Component at the Capsid-Tegument Interface of Herpes Simplex Virus 1

## Supplemental material

**Files in this Data Supplement:**

- Supplemental file 1 -

  Fig. S1 (Primary EM images and reconstructions.)

  PDF, 8.0M
