# Supplementary material for: The Large Tegument Protein pUL36 Is Essential for Formation of the Capsid Vertex-Specific Component at the Capsid-Tegument Interface of Herpes Simplex Virus 1
Source: J Virol. 2014 Nov 19;89(3):1502–11. doi: 10.1128/JVI.02887-14 (PMC4300765; doi:10.1128/JVI.02887-14)
Supplement: Supplemental material [file JVI.02887-14_zjv999099990so1.pdf]

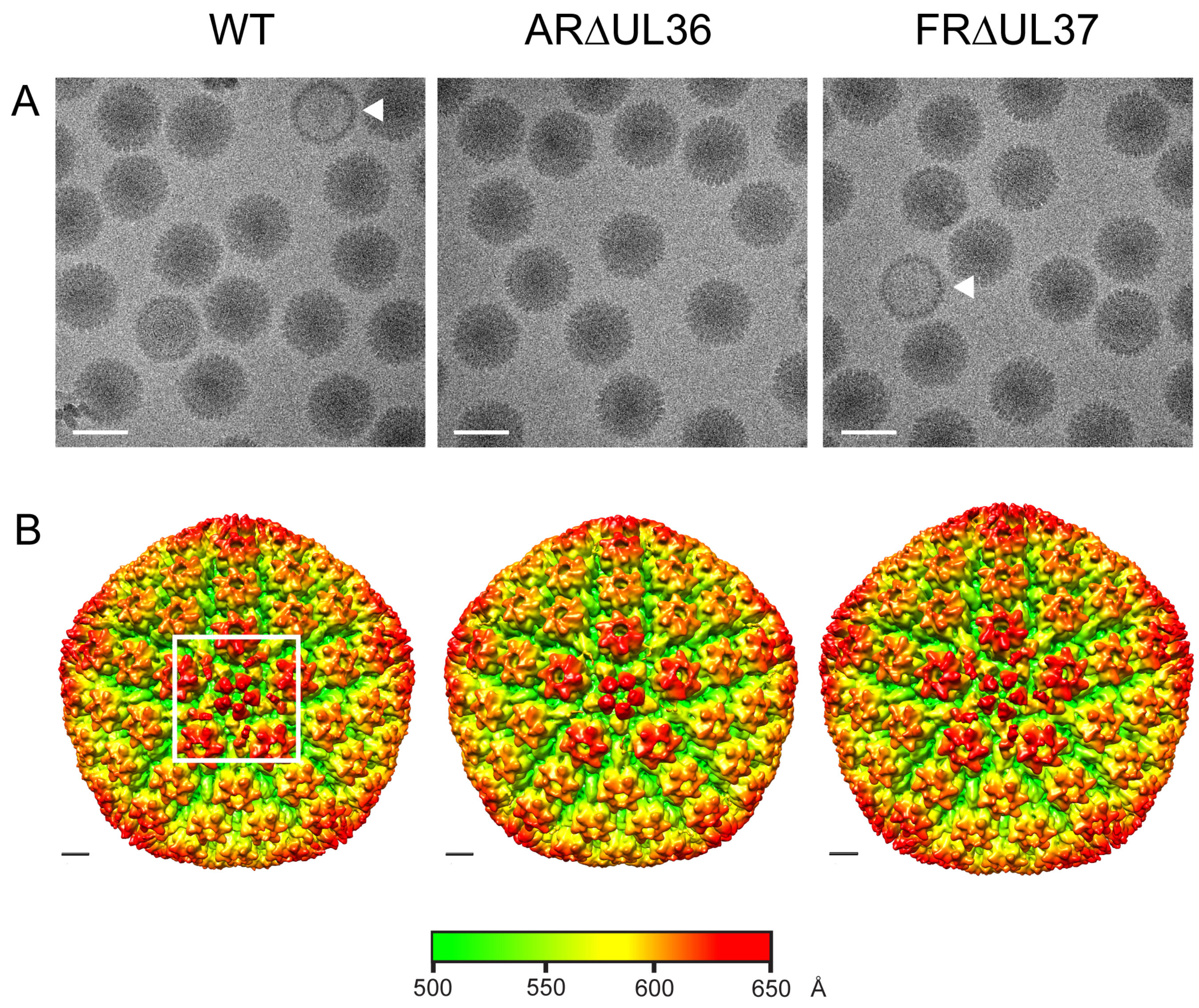

Supplemental FIG 1 3D structures of HSV-1 WT and mutant virus capsids. (A) Representative images of ice-embedded cytoplasmic C-capsids prepared from WT-, ARΔUL36- and FRΔUL37 -infected cells. Capsids lacking internal DNA are indicated by white arrowheads. Scale bar = 100 nm (B) Radially colored surface representation of the WT and mutant capsids. The white box in the left-hand image demarcates the capsid region shown enlarged in Figs 1, 3 and 4. The color bar indicates the radial distance from the center of each capsid. Scale bar = 100 Å.

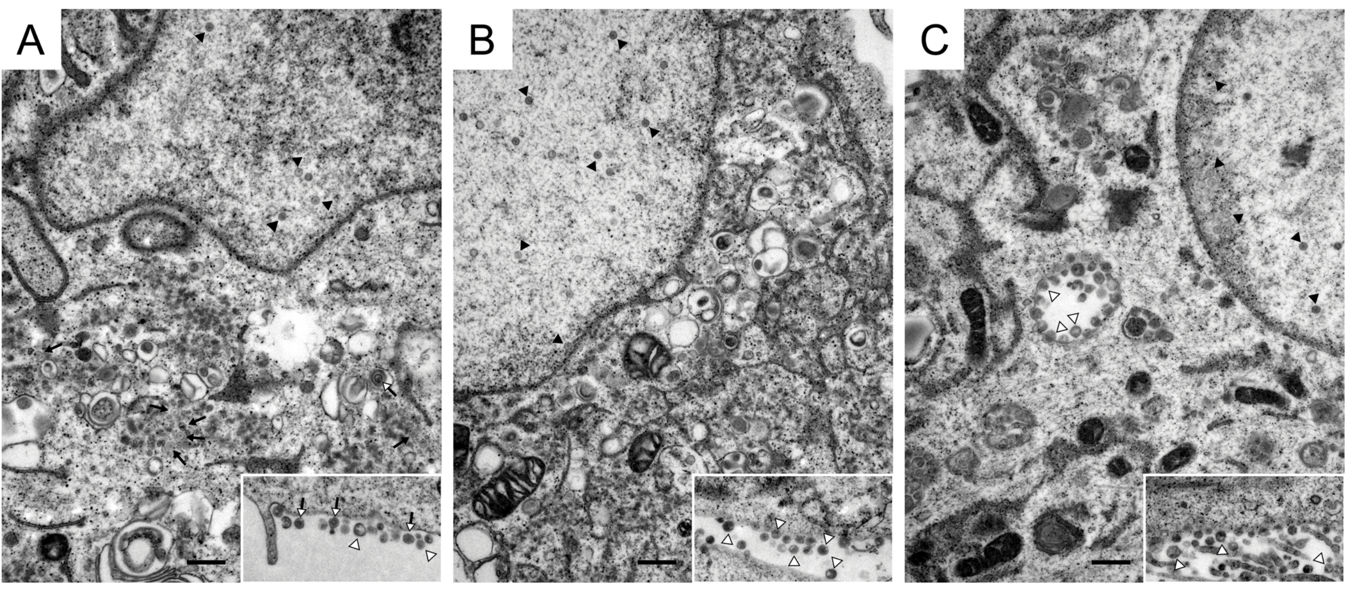

Supplemental FIG 2 Transmission EM analysis of cells infected with the UL34 minus mutant, vRR1072. Micrographs show UL34 expressing Vero (143/1099E) cells (41) (A), control Vero cells (B), and BHK cells (C) infected with vRR1072 for 20 h. Both nuclear (black arrowhead) and cytoplasmic (black arrow) capsids are indicated in A but no capsids were present outside the nucleus in B or C. Inserts show viral particles at the cell surface, comprising a mixture of virions (white arrows) and L-particles (which lack capsids (19, 22); white arrowheads) in A but only L-particles in B and C. Nuc = nucleus, Cyt = cytoplasm. Scale bar = 500 nm.

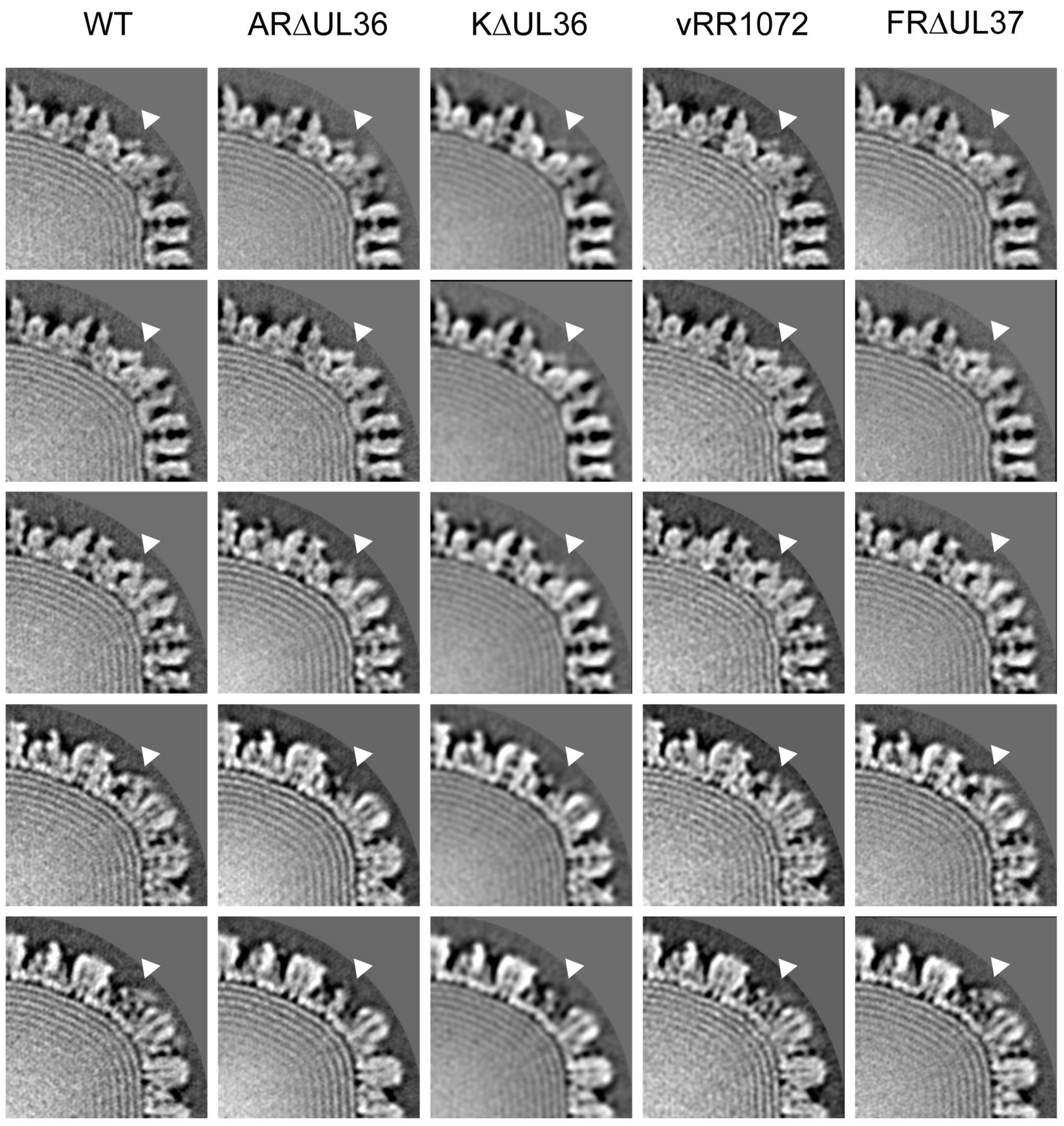

Supplemental FIG 3 CVSC occupancy on WT and mutant capsids. Sequential sections are taken at 8 Å intervals from the centre section of cytoplasmic C-capsids prepared from WT-, ARΔUL36-, KΔUL36-, vRR1072- and FRΔUL37-infected cells. The locations of the CVSC densities are indicated by white arrowheads.
